# Identification of Fungal Pathogens to Control Postharvest Passion Fruit (*Passiflora edulis*) Decays and Multi-Omics Comparative Pathway Analysis Reveals Purple Is More Resistant to Pathogens than a Yellow Cultivar

**DOI:** 10.3390/jof7100879

**Published:** 2021-10-19

**Authors:** Hafiz Muhammad Rizwan, Lin Zhimin, Wiwiek Harsonowati, Abdul Waheed, Yang Qiang, Ahmed Fathy Yousef, Nigarish Munir, Xiaoxia Wei, Sandra S. Scholz, Michael Reichelt, Ralf Oelmüller, Faxing Chen

**Affiliations:** 1 College of Horticulture, Fujian Agriculture and Forestry University, Fuzhou 350002, China; chrizwan51@gmail.com (H.M.R.); 1200305020@fafu.edu.cn (Y.Q.); Ahmedfathy201161@yahoo.com (A.F.Y.); nigarish.munir@yahoo.com (N.M.); ralf.oelmueller@uni-jena.de (R.O.); 2 Institute of Biotechnology, Fujian Academy of Agricultural Sciences, Fuzhou 350002, China; lzm@fjage.org; 3 Department of Bioresource Science, College of Agriculture, Ibaraki University, 3-21-1 Chuuo, Ami, Inashiki, Ibaraki 300-0393, Japan; w_harsonowati@yahoo.com; 4 Key Laboratory for Bio Pesticide and Chemical Biology, Ministry of Education, Fujian Agriculture and Forestry University, Fuzhou 350002, China; Waheed90539@gmail.com; 5 Department of Horticulture, College of Agriculture, University of Al-Azhar (Branch Assiut), Assiut 71524, Egypt; 6 Fruit Research Institute, Fujian Academy of Agricultural Sciences, Fuzhou 350002, China; zhw7782352@sina.com; 7 Matthias Schleiden Institute, Plant Physiology, Friedrich-Schiller-University Jena, Dornburger Str. 159, 07743 Jena, Germany; s.scholz@uni-jena.de; 8 Department of Biochemistry, Max Planck Institute for Chemical Ecology, Hans-Knöll-Str. 8, 07745 Jena, Germany; reichelt@ice.mpg.de

**Keywords:** disease management, ITS-rDNA sequence, metabolic profiling, pathogenic fungi, transcriptomics

## Abstract

Production of passion fruit (*Passiflora edulis*) is restricted by postharvest decay, which limits the storage period. We isolated, identified, and characterized fungal pathogens causing decay in two passion fruit cultivars during two fruit seasons in China. Morphological characteristics and nucleotide sequences of ITS-rDNA regions identified eighteen isolates, which were pathogenic on yellow and purple fruit. *Fusarium kyushuense*, *Fusarium concentricum*, *Colletotrichum truncatum*, and *Alternaria alternata* were the most aggressive species. Visible inspections and comparative analysis of the disease incidences demonstrated that wounded and non-wounded yellow fruit were more susceptible to the pathogens than the purple fruit. Purple cultivar showed higher expression levels of defense-related genes through expression and metabolic profiling, as well as significantly higher levels of their biosynthesis pathways. We also found fungi with potential beneficial features for the quality of fruits. Our transcriptomic and metabolomics data provide a basis to identify potential targets to improve the pathogen resistance of the susceptible yellow cultivar. The identified fungi and affected features of the fruit of both cultivars provide important information for the control of pathogens in passion fruit industry and postharvest storage.

## 1. Introduction

Passion fruit (*Passiflora edulis*) belongs to the *Passifloraceae* family and is a perennial evergreen climbing vine with more than 500 species. The plant is widely cultivated throughout tropical and subtropical regions of the world. Brazil is one of the largest producers of passion fruit [1]. From the estimated 1.46 million tons of global production in 2017, 1 million tons came from Brazil [2]. Passion fruit is usually consumed as fruit juice; it is rich in nutrients with pleasing and diverse aromas, and the fresh fruit is used as raw material in the beverage industry [3]. The passion fruit is also rich in flavonoids, alkaloids, and other bioactive compounds used for traditional medicines in several countries, such as flowers for bronchitis and cough, leaf extracts for insomnia, anxiety, and alcoholism, and seed oil for oil massages and as a lubricant [4]. Recently, two cultivars, the acidic purple passion fruit (*P. edulis* cv. Sims) and the sweeter yellow passion fruit (*P. edulis* cv. flavicarpa), have become important commercial fruit and are growing at a large scale in different provinces of China, especially in Guangdong, Fujian, Yunnan, Guangxi, and Taiwan [5]. The passion fruit industry and market are constantly evolving worldwide due to the increasing demand of the active ingredients in the fruit and the health benefits for consumers.

Although many factors affect the fruit quality, such as the selection of the variety, the cultivation system, climatic conditions, harvesting procedures and times [6], the market suffers most under the rapid decomposition of the harvested fruit, which cannot be stored for longer periods at room temperatures. The enormous postharvest losses between harvest and consumption are caused by the high susceptibility of the fruit to pathogen infections, which can only be counteracted by careful handling, packaging, and transportation, e.g., at lower temperatures [7]. In particular, physical damage, softening, water loss, and shriveling lead to fruit decay and deterioration, which results in huge economic loss [8]. According to the Food and Agriculture Organization (FAO), the global average loss due to food postharvest losses are about 29% in highly developed countries and about 38% in developed Asian, African, Latin American, and Southeast Asian countries [9], and these losses are even higher for horticultural crops and exotic fruit [10]. Microbial infections are the main cause for postharvest losses worldwide and the major post-harvest diseases are green and blue mold caused by *Penicillium digitatum* and *P. italicum* [11], brown rot by *Monilinia fructicola*, *Alternaria* black spot by *Alternaria alternata*, Rhizopus rot by *Rhizopus stolonifer* [12], and anthracnose by *Colletotrichum brevisporum*, *C. boninense*, and *C. brevisporum* [13].

Previous reports from different countries identified different pathogens that cause postharvest passion fruit decay, such as *C. brevisporum* in China and Japan [14,15], *C. gloeosporioides* in Brazil [16], *Lasiodiplodia theobromae* in China [17], and *Fusarium semitectum* in Brazil [18], as well as *Phytophthora nicotianae* var. *parasitica*, *P. drechsleri,* and *C. gloeosporioides* sensu lato in Colombia [19]. These findings suggest that the pathogens that caused decay could be differ in different areas of the world. Molecular identification of fungal pathogens usually relies on sequencing the nuclear ribosomal internal transcribed spacer (ITS) region [20,21,22,23,24], which is often complemented by morphological and physiological studies. Since passion fruit has become commercially important in China, the industry searches intensively for tools to restrict postharvest decay. An important aspect in this scenario is the identification of the fungal pathogen species that cause the disease. Therefore, the first objectives were (i) the isolation, identification, and morphological characterization of the pathogenic and non-pathogenic fungal species associated with the postharvest passion fruit decay; (ii) the characterization of the pathogenicity of the fungal pathogens, which are living on the fruit of the yellow and purple cultivars; and (iii) the confirmation of the identified pathogens through molecular analysis. Summed up, our findings showed that the identified pathogens propagate faster on the fruit of the yellow cultivar. Therefore, we performed a multi-omics approach with the peels of the yellow and purple fruit to understand why the purple fruit are more resistant to infections with those pathogens, which were found on both types of fruits. 

## 2. Materials and Methods

### 2.1. Fruit Sample Collection

The experiments were carried out during 2019–2020 at the Institute of Subtropical Fruit, Fujian Agriculture and Forestry University, China. The infected fruits (thirty fruits from each cultivar with three replications) of the commercial passion fruit cultivars, yellow (*P. edulis*. Flavicarpa cv Huangjin) and purple (*P. edulis*. Sims cv Tainong), were collected from private orchards located in Fujian province, China (23°48′35.2″ N and 117°07′08.1″ E). Thereafter, fruit were moist incubated at room temperature (25 ± 2 °C) for 2 weeks by placing them in plastic containers, covered with lids and moist towel paper to maintain the relative humidity (RH) for pathogen growth and development. The collected fruits showed decay symptoms within 2 weeks.

### 2.2. Fungal Pathogens Isolation

The infected tissues were cut into 3–5 mm pieces from the symptomatic fruit and surface-sterilized by immersion in 75% (*v*/*v*) ethanol for 30 s, followed by 1% sodium hypochlorite (NaClO) for 3 min, rinsed 3 times with sterile distilled water, and dried on sterilized tissue paper. The surface-sterilized tissues were then cultured on 90 mm Petri dishes containing potato dextrose agar (PDA) medium (38 g PDA in 1 L distilled water) amended with 100 mg L^−1^ ampicillin to prevent bacterial contamination, and incubated for 12 h photoperiods at 25 ± 2 °C for 5–7 d. Emerging colonies were then transferred many times to fresh PDA plates by the hyphal tip method until pure cultures were obtained. Pure cultures were further grown on PDA medium at 25 ± 2 °C for morphological characterization, DNA extraction, and pathogenicity tests. All isolates were maintained and stored in 20% glycerol at −80 °C until use.

### 2.3. Morphological Identification of Fungal Isolates

The morphology of fungal isolates was studied macroscopically by observing the colony features (shape, color, size). For the microscopic study, the fungi were grown on PDA medium at 25 ± 2 °C for 7–10 d. Small PDA pieces (2 × 2 × 2 mm) were sandwiched between two 18 × 18 mm cover glasses and placed on water agar plates to provide humidity. A small spore fragment was taken from the fresh sprouting culture by sterilized needle and distributed on the edge of the surface of the small PDA pieces, before incubation at 25 ± 2 °C for 2 weeks. The glass covers were carefully mounted on 76 × 26 mm micro glass slides after sufficient growth of the cultures. The conidia were observed under an optical microscope with a digital camera at 40× magnification (Phenix, BMC300, Jiangxi, China).

### 2.4. Molecular Identification of Fungal Isolates

Fungal isolates were grown on PDA medium at 25 ± 2 °C for 2 weeks, to confirm the morphological features. The fresh mycelia from each isolate were scraped directly from the plates with sterilized glass slides and transferred to 1.5 mL Eppendorf tubes for genomic DNA extraction using the PREPMAN Ultra Sample Preparation Reagent (Cat# 4318930, ThermoFisher, Applied Biosystems™, Warrington, UK) according to the manufacturer’s protocol. The partial 18S small subunit (SSU), internal transcribed spacer (ITS) 1–5.8 S-ITS2 region, and partial 28S large subunit (LSU) were amplified from the genomic DNA by polymerase chain reaction (PCR) with universal primers ITS1F: 5′-CTTGGTCATTTAGAGGA AGTAA-3′ and ITS4-R: 5′-TCCTCCGCTTATTGAT ATGC-3′, respectively [25]. PCR amplification was conducted in a 50 µL reaction volume containing 2 µL genomic DNA (100 ng), 2.5 µL of each primer (100 µM), 25 µL of 2 Hieff Canace^®^ Gold PCR Master Mix (Cat# 10149ES08, Yeasen Bio, Shanghai, China), and sterilized Milli-Q water. PCR was carried out by using a T100^TM^ Thermal Cycler (Bio-Rad, Singapore) with the following conditions: 94 °C for 4 min; followed by 35 cycles for 30 s at 94 °C; 55 s annealing at 52 °C, 2 min at 72 °C; and a final extension for 10 min at 72 °C; holding at 12 °C. The PCR products were analyzed by running the sample on a 1.5% agarose gel in 1× TAE (Tris-acetate-EDTA) buffer and bands were visualized in UV transilluminator (Peiqing, Model: JS-680D, shanghai, China). The PCR products were purified using SanPrep Column PCR Product Purification Kit No: B518141 and were custom sequenced at Sangon Biotech (Sangon Bio, shanghai, China). The obtained sequences were assembled to contig by using ChromasPro software 2.1.8 (Technelysium Pty Ltd., Brisbane, Australia). The obtained sequences were compared using the National Center for Biotechnology Information (NCBI) online nucleotide basic local alignment search tool (BLAST) database for closely related taxa (http://www.ncbi.nlm.nih.gov/blast/ accessed on 5 August 2020). A name was allotted to the selected isolate, when the BLAST hits were ≥ 98% for the top three matchings with the same species [22]. GenBank sequences were submitted to NCBI GenBank database under accession numbers MW880893–MW880918.

### 2.5. Phylogenetic Analysis

For phylogenetic analysis, the sequence data obtained for the 26 isolates were used for sequence similarity search in the GenBank (Table S1), and the results edited with the MEGA-X software 10.1.8. The neighbor-joining method was used to infer the evolutionary relationship of the fugal isolates in this phylogenetic tree and bootstrapped through 1000 replications to determine the percentage among clades. 

### 2.6. Pathogenicity Test

The 26 isolates from 12 morphotypes identified in this study were tested on the healthy yellow (*P. edulis*. Flavicarpa cv Huangjin) and purple (*P. edulis* Sims cv Tainong) passion fruit cultivars for their pathogenicity by two ways. (i) Wounded fruit were infected with 1 mL of a 1 × 10^6^ conidia mL^−1^ suspension, and (ii) non-wounded intact fruit by using 5-mm mycelial plugs. Spore suspensions were prepared by incubating the isolates on PDA media at 25 ± 2 °C, in light–dark (18–6 h) cycles for 7–10 d. The inoculum was prepared by flooding the cultures with 10 mL distilled water and the surface was slightly scraped with sterilized cell spreader before the conidial suspensions were sieved by using double layers of muslin cloth. The conidial concentration was examined and adjusted to 1 × 10^6^ conidia mL^−1^ by using a hemocytometer. Fruit were washed with tap water, surface sterilized by soaking in 75% (*v*/*v*) ethanol for 30 s, followed by 2% NaClO solution for 3 min, rinsed 3 times with sterile distilled water, and air-dried for approximately 30 min in a laminar flow chamber. All fruit were wounded with a sterile needle about 3 mm depth in the equatorial zone and 10 µL conidial suspensions (1 × 10^6^ conidia mL^−1^) were pipetted on the wounded spots, while control fruit were treated with sterilized distilled water. Mycelia plugs around 5 mm in diameter from each isolate were inoculated on the equatorial zone with PDA plug as control for non-wounded fruits. The inoculated fruit were moist incubated by placing them into plastic containers on wet paper at 25 ± 2 °C, light-dark (18–6 h) cycle and 80% RH. Three wounded and three non-wounded fruit were inoculated per cultivar for each isolate and the experiment was repeated twice. Fruits were observed and photographed on the 4th, 8th, and 12th day post-inoculation (dpi) to record the disease incidence and the lesion diameter. After 12 dpi, the fruit were cut through the center of lesions to observe the symptoms and lesions. The fungi were re-isolated from the resulted lesion margins of the inoculated fruit and cultured on fresh PDA plates, and then re-identified by comparing their morphological and microscopic features with those of the original isolates.

### 2.7. Phytohormones Analysis

Phytohormones were extracted from the peels of the fruit of the yellow and purple passion fruit cultivars. Frozen samples were homogenized for 30 s at 1000 strokes per minute in a 2010 Geno/Grinder^®^ (SPEX SamplePrep, Stanmore, UK) and mixed with 1 mL methanol containing 40 µg/L^−1^ of D_6_-JA (HPC Standards GmbH, Cunnersdorf, Germany), D_6_-ABA (Toronto Research Chemicals, Toronto, Canada), D_4_-SA (Santa Cruz Biotechnology, TX, U.S.A) and 8 µg L^−1^ of D_6_-JA-Ile (HPC Standards GmbH, Cunnersdorf, Germany). All samples were shaken for 30 min at 4 °C and centrifuged at 17,900× *g* for 20 min at 4 °C. The supernatants were collected and the sample re-extracted with 500 μL methanol. The combined supernatants were evaporated to dryness at 30 °C using a vacuum concentrator (Eppendorf, Wesseling, Germany). Residues were re-suspended in 200 μL methanol and centrifuged at 17,900× *g* for 10 min. The supernatants were collected and measured with the QTRAT6500 LC-MS/MS system (AB Sciex, Darmstadt, Germany) as previously described. Since it was observed that both the D_6_-labeled JA and JA-Ile contained 40% of the corresponding D_5_-labeled compounds, both peaks were combined for analysis.

### 2.8. RNA Extraction, cDNA Library Construction, and Transcriptome Sequencing

Total RNA was extracted and purified from yellow and purple fruit peel samples following the manufacturer’s instructions using Tiangen Kits (Tiangen, China). The RNA quality was examined by using agarose gel electrophoresis (1%) and 2100 Bioanalyzer (Agilent Technologies, CA, USA). The sequencing libraries were generated using a NEBNext^®^ Ultra™ RNA Library Prep Kit for Illumina^®^ (NEB, Boston, MA, USA) following the manufacturer’s instructions. In order to select cDNA fragments of preferentially 240 bp in length, the library fragments were purified with AMPure XP system (Beckman Coulter, Beverly, MA, USA). The clustering of the index-coded samples was performed on a cBot Cluster Generation System using TruSeq PE Cluster Kit v3-cBot-HS (Illumina, San Diego, CA, USA) according to the manufacturer’s recommendation. After cluster generation, the library preparations were sequenced on an Illumina HiSeq 2000 platform and paired-end reads were generated (Biomarker, Technologies Corporation, Bejing, China). FASTQ format was used to store raw reads. The high quality reads (clean reads) were obtained by removing the low-quality reads, reads containing ploy-N and adapter sequences from the raw reads processed through in-house Perl scripts. At the same time, Q20, Q30, GC-content and sequence duplication level of the clean data were calculated. Trinity version 2.5.1 was used to assemble the transcriptome using the left.fq and right.fq files, with the min kmer cov parameter set as 2 by default and all the other parameters specified as defaults. 

### 2.9. Gene Function Annotation, Expression, and Pathway Analysis

The assembled Unigenes sequences were BLASTed in NCBI non-redundant protein sequences (NR); Protein family (Pfam); Clusters of Orthologous Groups of proteins (KOG/COG/eggNOG); Swiss-Prot (a manually annotated and reviewed protein sequence database); Kyoto Encyclopedia of Genes and Genomes (KEGG); and Gene Ontology (GO) databases using BLAST version 2.2.31. The predicted UniGene amino acid sequences were compared with the Pfam database using HMMER software version 2.2.31, to obtain the annotation information. Gene expression level of all the samples were estimated by mapping the clean reads to the Trinity transcripts assembly, using bowtie2 combined with RSEM version 1.2.1. The read count for each gene was obtained from the mapping results. The FPKM (Fragments Per Kilobase of transcript per Million) value was used to indicate the expression abundance. Prior to differential gene expression analysis for each sequenced library, the read counts were adjusted by EBSeq program package through empirical Bayesian approach. Differential expression analysis of two samples was performed using the EBSeq R package. *p*-value was adjusted using *q*-value < 0.005 and |log_2_ (fold change)| > 1 was set as the threshold for significantly differential expression. GO enrichment analysis of the differentially expressed genes (DEGs) was identified by comparing the reads of the purple with the yellow cultivar and was implemented by the topGO R packages version 2.28.0 based on the Kolmogorov–Smirnov test. KEGG enrichment analysis was performed using KOBAS version 2.0.12. After identifying the putative defense-related candidate genes, the respective pathways were analyzed using the KEGG Mapper tool of the Kyoto Encyclopedia of Genes and Genomes (https://www.genome.jp/kegg/tool/map_pathway2.html accessed on 15 May 2021).

### 2.10. Non-Target Metabolite Analysis by LC-ESI-Q-ToF-MS

For the metabolite profiles, the fruit from the purple and yellow cultivars were harvested, the peels were separated from the fruit, frozen in liquid nitrogen, ground with a mortar and pestle and lyophilized. The obtained powder was used for LC-ESI-Q-ToF-MS analyses. A total of 10 mg of ground peel samples was extracted with 1 mL methanol (from three independent fruit harvests each). For non-target analysis, ultra-high-performance liquid chromatography–electrospray ionization–high resolution mass spectrometry (UHPLC–ESI–HRMS) was performed with a Dionex Ultimate 3000 series UHPLC (Thermo Fisher Scientific, Waltham, MA, USA) and a Bruker timsToF mass spectrometer (Bruker Daltonics, Bremen, Germany). UHPLC was used applying a Zorbax Eclipse XDB-C18 column (100 mm × 2.1 mm, 1.8 µm, Agilent Technologies, Waldbronn, Germany) with a solvent system of 0.1% formic acid (A) and acetonitrile (B) at a flow rate of 0.3 mL min^−1^. The elution profile was the following: 0 to 0.5 min, 5% B; 0.5 to 11.0 min, 5% to 60% B in A; 11.0 to 11.1 min, 60% to 100% B, 11.1 to 12.0 min, 100% B and 12.1 to 15.0 min 5% B. Electrospray ionization (ESI) in negative/positive ionization mode was used for the coupling of LC to MS. The mass spectrometer parameters were set as follows: capillary voltage 4.5 KV/3.5 KV, end plate offset of 500 V, nebulizer pressure 2.8 bar, nitrogen at 280 °C at a flow rate of 8 L min^−1^ as drying gas. Acquisition was achieved at 12 Hz with a mass range from m/z 50 to 1500. At the beginning of each chromatographic analysis, 10 µL of a sodium formate-isopropanol solution [10 mM solution of NaOH in 50/50 (*v*/*v*%) isopropanol water containing 0.2% formic acid] was injected into the dead volume of the sample injection for recalibration of the mass spectrometer using the expected cluster ion m/z values.

Data processing: the LC-Q-ToF-MS raw data were recalibrated and then processed with MetaboScape software (Bruker Daltonik GmbH, Bremen, Germany). Automated peak picking and alignment were done within a retention time between 0.4 and 11 min, intensity threshold 500, and minimum occurrence in three out of six samples. Feature intensities were normalized by the fresh weight of the plant material used for extraction. Missing values were replaced by 20. Feature groups, potentially representing single metabolites, were reduced to one bucket by the MetaboScape software to represent the respective metabolite in later analysis. The following commercial standards were used to verify the identity of the identified compounds by match of retention time and mass spectrum: D-(+)-Glucose (Sigma-Aldrich), D-(+)-Sucrose (Sigma-Aldrich), L-glutamic acid (Sigma-Aldrich), L-proline (Duchefa), DL-malic acid (Sigma-Aldrich), citric acid (Carl Roth), glutathione reduced form (Sigma-Aldrich), L-tyrosine (Duchefa), adenosine (Sigma-Aldrich), L-phenylalanine (Duchefa), L-glutamine (Sigma-Aldrich), L-(+)-ascorbic acid (Carl Roth), adenosine (Sigma-Aldrich), cyanidin-3-O-glucoside (TransMIT, Gießen, Germany), epicatechin (Sigma-Aldrich), catechin (Sigma-Aldrich), gallocatechin (Cayman Chemical). Citrusin A/hyuganoside III and prunasin were tentatively identified by comparison to literature data [26] (compound numbers 13, 14, and 3 in this reference), and prunasin-rhamnoside according to Farag, Otify [27] (compound number 10 in this reference).

### 2.11. Statistical Analysis

All statistical analyses were performed using SPSS version 19.0 (SPSS, IBM, Armonk, NY, USA) and one-way analysis of variance (ANOVA) was applied to the lesion data of the infected passion fruit from each pathogen. The differences were determined by using Tukey’s test and were considered statistically significant if *p* < 0.05.

## 3. Results

### 3.1. Postharvest Decay Symptoms in Passion Fruits

**Figure 1:**
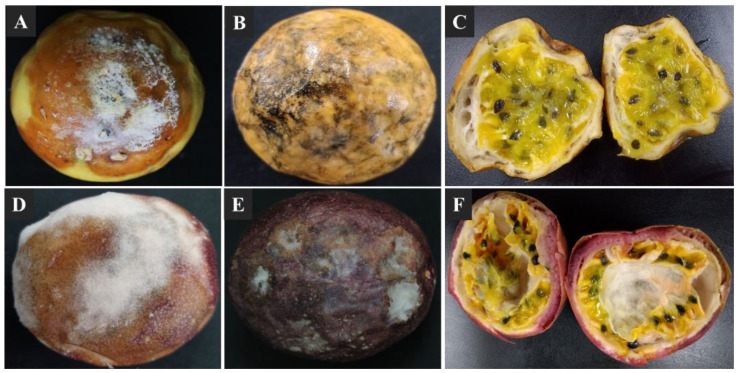
Postharvest decay symptoms on yellow and purple passion fruit; (**A**,**D**) visible soft decay symptoms causing soggy fruit; (**B**,**E**) visible dry and irregular decay symptoms; (**C**,**F**) cross-sections of decayed passion fruit.

Soft and dry visible decay symptoms were observed on the surface of passion fruit within 2 weeks of storage at 25 ± 2 °C. The 30–40% visible soft-decay lesions were observed on the fruit surface and characterized by a white to yellowish-light brown skin color, which expand to the whole fruit surface making a soggy fruit (Figure 1A,D). The 10–20% visible dry decay lesions were observed on fruit surface and characterized by gray to brownish-black with irregular sunken cavities, which expand to the whole fruit surface under suitable environmental conditions (Figure 1B,E). When the decay fruit were cross sectioned, the affected peels and fleshes seemed softer, disorganized, water-saturated, and darker in color (Figure 1C,F).

### 3.2. Fungus Isolates from Infected Passion Fruit

**Figure 2:**
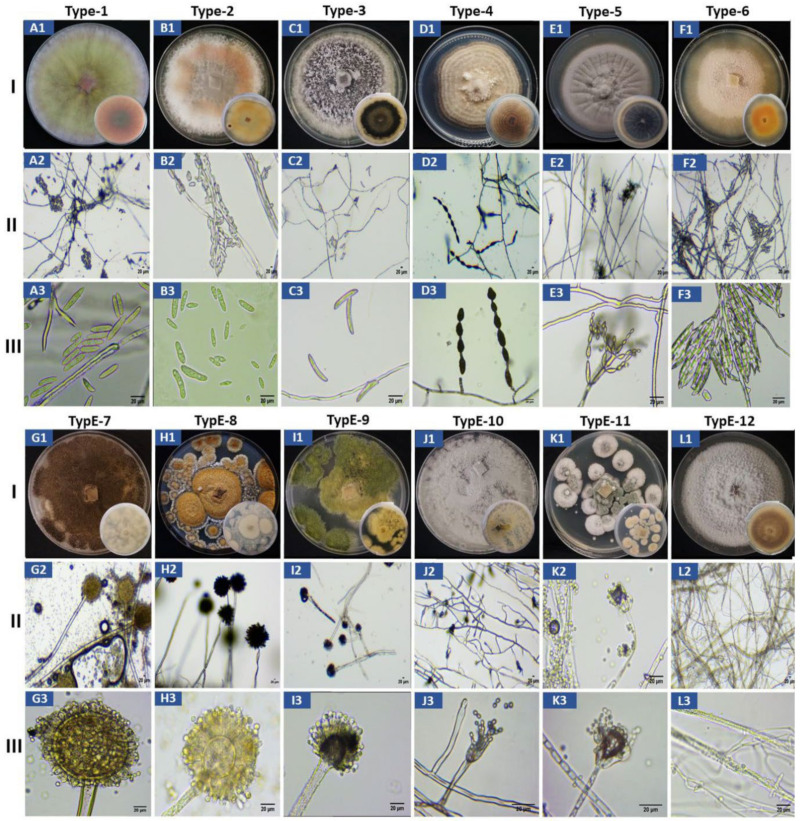
Morphological characterization of passion fruit isolates type-1 to type-12; I (**A1**–**L1**) colony morphology on PDA media, II (**A2**–**L2**) thread like structure showing hyphae and mycelium and III (**A3**–**L3**) conidia. Type-1 (**A1**–**A3**) = *Fusarium kyushuense*; type-2 (**B1**–**B3**) = *Fusarium concentricum*; type-3 (**C1**–**C3**) = *Colletotrichum truncatum*; type-4 (**D1**–**D3**) = *Alternaria alternata*; type-5 (**E1**–**E3**) = *Cladosporium tenuissimum*; type-6 (**F1**–**F3**) = *Fusarium equiseti*; type-7 (**G1**–**G3**) = *Aspergillus aculeatus*; type-8 (**H1**–**H3**) = *Aspergillus europaeus*; type-9 (**I1**–**I3**) = *Aspergillus flavus*; type-10 (**J1**–**J3**) = *Penicillium chermesinum*; type-11 (**K1**–**K3**) = *Penicillium paxilli*; type-12 (**L1**–**L3**) = *Microdochium phragmitis*. (Bar = 20 µm).

A total of 26 fungal isolates (13 from yellow and 13 from purple passion fruit) were collected and purified from the infected passion fruit (Figure S1). The isolates from yellow passion fruit were named YPF-1 toYPF-13 and those from the purple passion fruit PPF-1 to PPF-13. Based on morphological inspections and growth parameters, the 26 isolates were initially categorized into 12 different morphotypes (Figure 2, Table 1), which later confirmed by internal transcribed spacer regions (ITS) sequencing. Based on ITS sequencing, 6 of the 26 isolates were obtained only once (= 6 morphotypes), 1 isolate was obtained twice, four or five times, respectively (= 3 morphotypes), and 3 isolates were obtained three times (= 3 morphotypes, Table 1). A morphotype contains isolates from only one or both cultivars. Interestingly, morphotypes 3, 6, 8, 9, 10, and 12 were only found on the yellow and morphotypes 4 and 11 on the purple cultivar. The isolates belong to different fungal species (spp.), 26.9% (7/26) *Fusarium* spp., 19.23% (5/26) *Alternaria* spp., 19.23% (5/26) *Aspergillus* spp., 15.38% (4/26) *Cladosporium* spp., 7.69% (2/26) *Colletotrichum* spp., 7.69% (2/26) *Penicillium* spp. and 3.85% (1/26) *Microdochium* spp. (Figure 2, Table 1).

### 3.3. Morphological Characterization of the 12 Morphotypes

The morphology of the 12 different types of fungi on PDA agar plates, the structure of the hyphae and mycelia as well as of the conidia are shown in Figure 2. A summary of all observations for each of the type of fungi is given in Table 2. Based on their growth rates, the form and color of the upper and lower sides of the colonies on PDA plates, the microscopic structure of the hyphae and mycelial organization, as well as the shape and morphological features of the conidia, we allocated the 12 different morphotypes to the following fungal species: *Fusarium kyushuense* (type-1), *F. concentricum* (type-2), *Colletotrichum truncatum* (type-3), *Alternaria alternata* (type-4), *Cladosporium tenuissimum* (type-5), *F. equiseti* (type-6), *Aspergillus aculeatus* (type-7), *A. europaeus* (type-8), *A. flavus* (type-9), *Penicillium chermesinum* (type-10), *P. paxilli* (type-11) and *Microdochium phragmitis* (type-12). Each allocation is based on comparison of the obtained lab information with literature data.

### 3.4. Molecular Characterization and Phylogenetic Analysis

**Figure 3:**
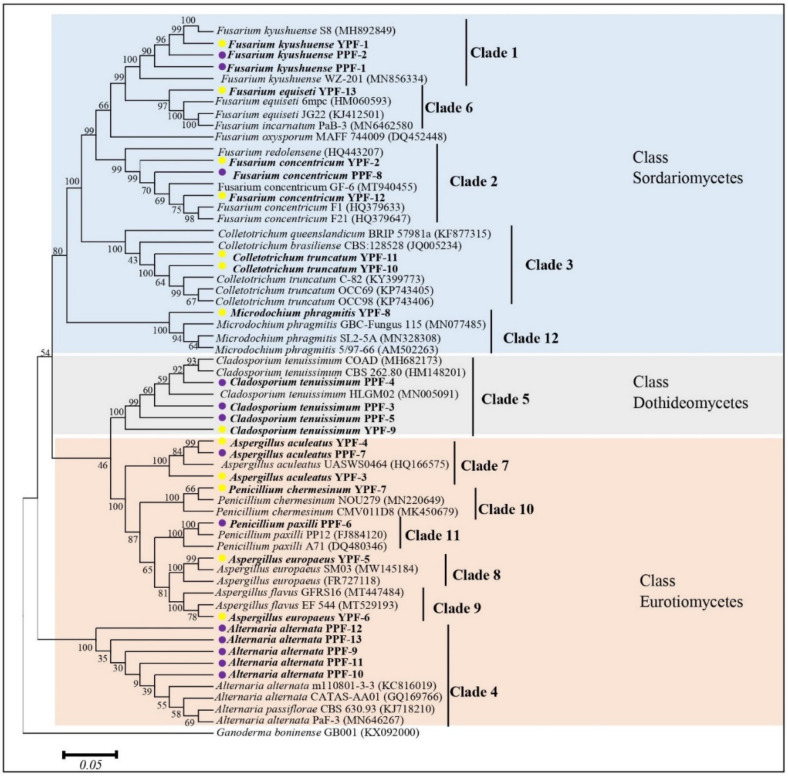
The evolutionary history of the twenty-six obtained isolates, presented in a phylogenetic tree on basis of neighbor joining and reference sequences from the NCBI GenBank database. The tree was constructed by analysis of ITS-rDNA sequences. Numbers at the nodes are the percentage of bootstrap support values of 1000 replicates. Ganoderma boninense GB001 (KX0920000) was used as an outgroup. The bar represents the 0.05 substitutions per nucleotide position and the isolates from the current study are presented in bold. Yellow and purple dots indicate the origin of the isolate.

The highly conserved ITS-rDNA regions (500–600 bp) were sequenced for the 26 isolates. A homology search with the BLASTN program at NCBI showed that the sequences from the isolates showed almost 100% similarity to the reference sequences (Table 3). Interestingly, the results of the molecular identification were identical to those obtained after morphological characterization of the isolates and explained in Table 2. Therefore, the twelve morphotypes represent twelve different fungal species. The ITS-rDNA sequences were used to study the relationship between the identified taxa (Figure 3). The sequences were clustered into three classes of Ascomycota and comprised of twelve clades from which each clade presents one of the twelve isolated fungal morphotype. Isolates of the morphotypes 1–3, 6, and 12 belong to the Sordariomycetes, morphotype 5 belongs to the Dothideomycetes, and morphotype 4 and 7–11 belong to the Eurotiomycetes. The GenBank accession numbers and details of reference sequences of the isolates used for the phylogenetic tree are presented in Table S1.

### 3.5. Pathogenicity Tests

**Figure 4:**
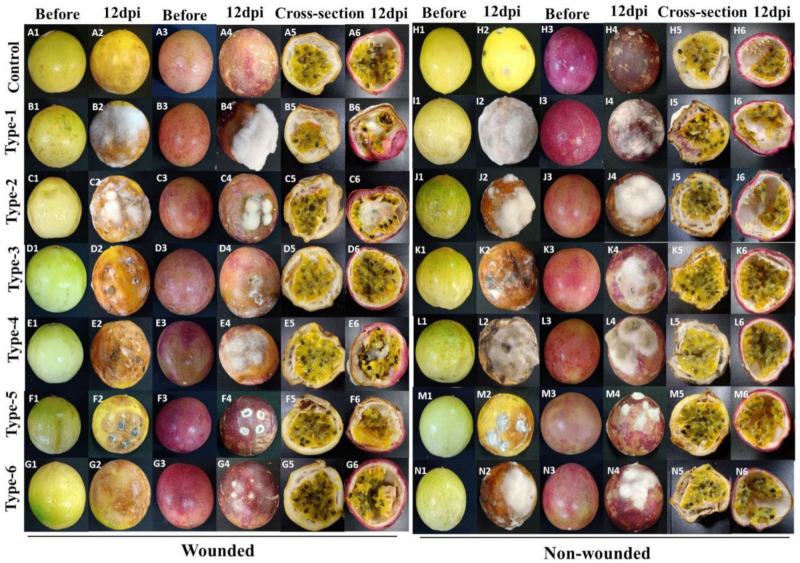
Pathogenicity of fungal isolates on two cultivars at 12 dpi. Type-1 (*Fusarium kyushuense*), type-2 (*Fusarium concentricum*), type-3 (*Colletotrichum truncatum*), type-4 (*Alternaria alternata*), type-5 (*Cladosporium tenuissimum*) and type-6 (*Fusarium equiseti*) isolates on yellow and purple passion fruits by wound (1 × 10^6^ conidia mL^−1^ suspension) and non-wounded (5 mm mycelial plug) methods. **A1**–**G6**) fruits treated with 1 × 10^6^ conidia mL^−1^ suspension of different fungal isolates using wound method, H1-N6) fruits treated with 5 mm mycelial plugs of different fungal isolates using non- wound method, **A1**–**A6** & **H1**–**H6**) fruits treated with water as control, **B1**–**B6** & **I1**–**I6**) fruits treated with Type-1 (*Fusarium kyushuense*) isolate, **C1**–**C6** & **J1**–**J6**) fruits treated with type-2 (*Fusarium concentricum*) isolate, **D1**–**D6** & **K1**–**K6**) fruits treated with type-3 (*Colletotrichum truncatum*) isolate, **E1**–**E6** & **L1**–**L6**) treated with type-4 (*Alternaria alternata*) isolate, **F1**–**F6** & **M1**–**M6**) fruits treated with type-5 (*Cladosporium tenuissimum*) isolate, **G1**–**G8** & **N1**–**N6**) fruits treated with type-6 (*Fusarium equiseti*) isolate.

**Figure 5:**
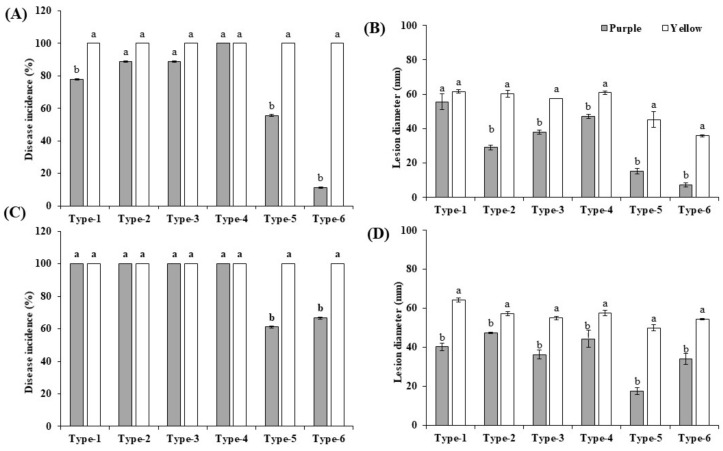
Determination of disease incidence (%), lesion diameter (mm) at12 dpi on yellow and purple passion fruits, inoculated by type-1 (*Fusarium kyushuense*), type-2 (*Fusarium concentricum*), type-3 (*Colletotrichum truncatum*), type-4 (*Alternaria alternata*), type-5 (*Cladosporium tenuissimum*), and type-6 (*Fusarium equiseti*) isolates. Results shown were obtained from (**A**,**B**) wounded fruits (1 × 10^6^ conidia mL^−1^ suspension) and from (**C**,**D**) non-wounded fruits (5 mm mycelial plug). Results are means ± standard error. Different letters (a/b) indicate significant differences between two cultivars.

Inoculation was performed with wounded and non-wounded methods, as described in Material and Methods. To inspect the virulence of the isolates, the disease development (disease incidence percentage and the lesion diameter) of the fruits was recorded 4, 8, and 12 dpi (only 12 dpi data are shown). The morphotypes 1 to 6 of *Fusarium*, *Colletotrichum*, *Alternaria,* and *Cladosporium* were pathogenic and produced the obvious rot symptoms on inoculated yellow and purple fruits in the assays with wounded and non-wounded material (Figure 4), while the morphotypes 7 to 12 of *Aspergillus*, *Penicillium*, and *Microdochium* did not, and were considered nonpathogenic. Development of the disease incidences and lesion diameters after infection of both cultivars with the six pathogenic isolates uncovered significant differences. After infection of wounded and non-wounded fruits, local necrosis and discoloration were clearly visible 4 days after infection and the disease development propagated over the entire surface of the fruits until the 12th day. Consistent with the disease phenotype, at the end of the experiment, the entire fruit surfaces were covered with the mycelia of the six pathogens (Figure 4(A2–G2,A4–D4,H2–N2,H4–N4)). The wounded fruits of both cultivars showed clearly stronger disease symptoms than the unwounded (Figure 4(A5–G6,H5–N6)), and this was observed for both cultivars. Cross sections 12 days after the infection demonstrated that the disease symptoms of the unwounded yellow fruits were much stronger than in the unwounded purple fruits (Figure 4(H5–N6)). Furthermore, symptom development in both wounded and unwounded yellow fruits occurred earlier than in purple fruits and this is reflected by higher disease indices and lesion diameters. Therefore, the yellow fruits are more susceptible to pathogen infections than the purple fruits. The control fruits did not develop any decay symptoms (Figure 4(A1–A6,H1–H6)). Comparison of the pathogenicity of the six fungi revealed that *F. kyushuense* (type-1), *F. concentricum* (type-2), *C. truncatum* (type-3), and *A. alternata* (type-4) species developed larger lesions than *C. tenuissimum* (type-5), and *F. equiseti* (type-6). The disease incidence percentage and lesion diameter (mm) of the pathogens are presented in Figure 5.

### 3.6. Phytohormones Levels in the Peels of the Fruit of the Yellow and Purple Cultivars

The different susceptibility of the fruit of the two cultivars to pathogen infections led us to investigate their defense capacities. Since the peels are an important defense barrier, we analyzed whether they differ in their defense-related hormone composition (Table 4). The SA and JA levels were only slightly, but not significantly higher in the purple peels, and the level of the active jasmonate, jasmonoyl-isoleucine (JA-Ile), was even higher in the yellow peels. Interestingly, *cis*-12-oxophytodienoic acid (*cis*-OPDA), the JA precursor, is 7-times higher in the yellow peels, although the overall level is quite low. Furthermore, the yellow peels contain more than twice as much ABA than the purple peels (Table 4). Taken together, the defense-related phytohormones levels are not higher in the purple peels. The difference suggests that the hormones have different functions in the two cultivars (cf. discussion).

### 3.7. Gene Function Annotation, Expression, and Pathway Analysis

**Figure 6:**
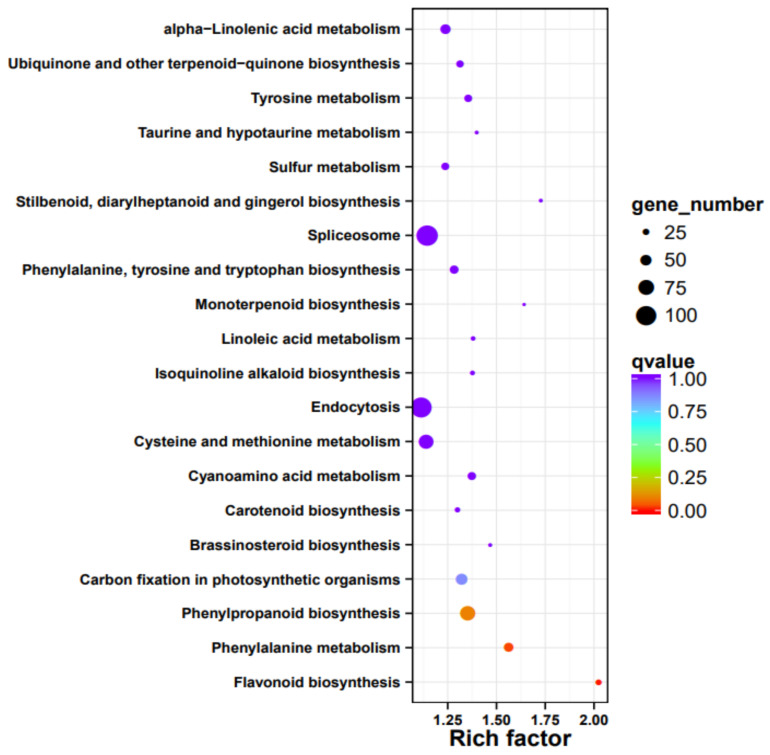
Pathway enrichment analysis based on the comparison of expression profiles from peels of purple vs. yellow fruits. Shown are the number of different sized dots (DEGs) of the top 20 regulated pathways. The rich factor on the x axes indicates the number of DEGs per pathway compared to the total number of genes in this pathway.

**Figure 7:**
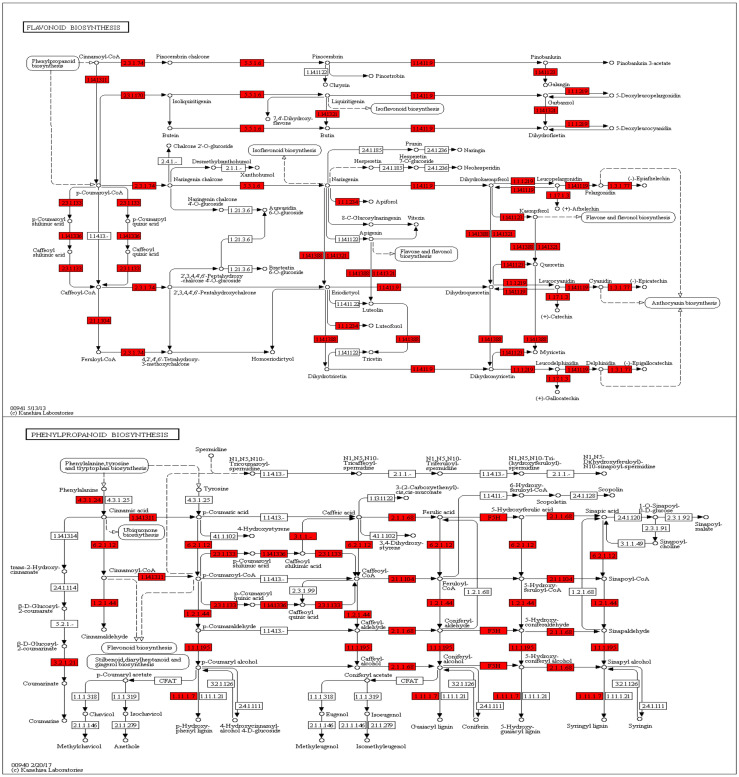
KEGG Mapper pathway analyses for enzymes involved in the flavonoid (**top**) and phenylpropanoid (**bottom**) biosynthesis. The genes for enzymes marked in red are higher expressed in the peels of the purple cultivar than the yellow cultivar. For experimental details, cf. Methods and Materials.

We isolated RNA form the peels of the two cultivars and compared the expression profiles. Overall, about 1/3 of all passion fruit genes are differentially expressed, and they represent almost all predicted biological processes (Table S2). An enrichment analysis identified those pathways, which contain the highest number of differently expressed genes (DEGs) in relation to the total gene numbers of the respective pathways (Figure 6). The biggest differences were observed for flavonoid biosynthesis (KEGG map00941), followed by DEGs categorized as “stilbenoid, diarylheptanoid, and gingerol biosynthesis” (KEGG map00945), “taurine and hypotaurine metabolism” (KEGG map00430), monoterpenoid biosynthesis (KEGG map00902), phenylalanine metabolism (KEGG map00360), brassinosteroid (KEGG map00905), and phenylpropanoid biosynthesis genes (KEGG map00940, Figure 6). Interestingly, defense-related and plant-pathogen-related genes are not enriched in one of the two cultivars, however, closer inspection uncovered that many defense-related genes with predicted antifungal activities are much stronger (>4-fold) up-regulated in the purple peels (Table 5). This includes genes for secondary metabolite biosynthesis, pathogenesis-related proteins, transporters, resistance and defense proteins, receptor kinases and defense signaling compounds, as well as regulators of redox homeostasis. As expected from the purple color, flavonoid biosynthesis genes, which include those for anthocyanin’s (cf. below), are stronger expressed in the purple peels (Table 5, Figure 6). This is clearly visible from the KEGG pathway analyses, which shows that many genes for enzymes for the phenylpropanoid and flavonoid biosynthesis are higher expressed in the purple peels (Figure 7). Taken together, the highly enriched defense-related secondary metabolites in the purple peels provide a better barrier against fungal attacks than the yellow peels. 

### 3.8. Differences in the Metabolite Profiles of Purple and Yellow Peels

The metabolite profile of both passion fruit varieties was analyzed; Table 6 shows the major identified compounds present in different concentrations in the two peels. The hypothesis that the color of the purple peels derives from anthocyanin was confirmed by identifying cyanidin-3-O-glucoside, and peonidin-3-O-glucoside as major component of their peels. In comparison, these two compounds were nearly absent in the peels of the yellow fruits. Additional antioxidants including catechin, epicatechin, and gallocatechin were found enriched in the purple peels. Several more flavonoids, which were not detectable in the yellow peels (for example peak 24, 25, Table 6), were detected in the purple ones. Their structure and function in the fruit is unknown and need to be analyzed in detail. In contrast to the previous observations, the flavonoid precursor phenylalanine itself was, with a five-fold concentration, more abundant in peels of the yellow fruits. Further detected components showed a significantly different content in the two varieties. Thus, the determined content of citric acid (peak 8, Table 6) was eight times higher in the purple peels, while other unknown compounds (peak 37, 38, Table 6) were enriched in the yellow fruits.

## 4. Discussion

Passion fruit has become an important commercial fruit with a high market value and large cultivation areas in China due to its favorable taste and phytonutrient ingredients [28,29,30]. However, the postharvest diseases limit its shelf life and market value and cause huge economic losses. The major postharvest decay symptoms and disease development depend strongly on the environmental and handling conditions. Previous studies identified the pathogens *Zythia versoniana* and *Coniella granati* as major causes for the dry decay [31] and *Aspergillus niger* for the soft decay [32]. Other reports identified the pathogenic *Colletotrichum* spp. [15,16], *Lasiodiplodia* spp. [17], and *Phytophthora* spp. [19] on the harvested fruits. Morphological analyses were described based on previous reports, and SSU, ITS, and LSU sequence data ultimately identified the fungal species (Table 3 and Table S1). A phylogenetic tree showed the relationship of the identified fungi to others (Figure 3). Pathogenicity studies showed that *F. kyushuense*, *F. concentricum*, *C. truncatum*, *A. alternata*, *C. tenuissimum,* and *F. equiseti* were pathogenic, while *A. aculeatus*, *A. europaeus*, *A. flavus*, *P. chermesinum*, *P. paxilli,* and *M. phragmitis* were not, since the re-infected fruit did not develop any detectable disease symptoms (Figure 4 and Figure 5). The six pathogenic fungi cause severe disease symptoms on wounded and unwounded yellow and purple passion fruit and have not yet been reported in the context of decay on harvested passion fruits. They belong to a well-known group of fungi involved in diseases on other host plants, such as *Fusarium* spp. on apple, citrus, banana, and blueberry, *Colletotrichum* spp. on strawberry, apple, citrus, and papaya, *Alternari*a spp. on apple, citrus, grapes, mango, pomegranate and *Cladosporium* spp. on grapes, pears, and raspberries. 

Thrane, Adler [33] reported that *F. kyushuense* strains produce the secondary metabolites aurofusarin, nivalenol, enniatin B and several aflatoxins. Therefore, uncontrolled growth of this fungal strain in the passion fruit could cause diseases during human consumption. On the other hand, *C. tenuissimum* produces Cladosporol A, a secondary metabolite, which exhibits antiproliferative properties in human colorectal cancer cells by modulating the expression of cell cycle genes [34]. Besides fungal products with negative effects on human consumption, *F. concentricum* and *F. equiseti* produce the well-known mycotoxin fusaric acid that plays an important role in plant pathology [35]. In addition, *C. truncatum* is a major cause for anthracnose in many crops including chili. Mishra, Mohanty [36] identified the chili *can-miRn37a* miRNA, which represses the expression of ethylene-response transcription factors and thus prevents fungal colonization and disease development. The large number of identified genes for ethylene-responsive transcription factors in the purple cultivar (Table 5) may contribute to the restriction of *C. truncatum* growth in the fruits. Furthermore, some of the fungal strains, which induce disease symptoms in infected fruit, may also have beneficial features in other organs, developmental stages, or microbial communities in the plant tissue. For instance, the *F. equiseti* strain GF19-1 is known as a plant-growth promoting fungus which induces systemic resistance via the SA pathway in Arabidopsis [37]. It shows pathogenic feature when inoculated alone to the passion fruit, however this might be restricted by other microbes, which are normally also present in the passion fruits.

Interestingly, one-third of the identified fungi do not cause pathogenic symptoms and they might function as beneficial symbionts. *Aspergillus aculeatus* has been reported as beneficial symbiont which confers salt, drought, heat and cadmium tolerance to the perennial ryegrass [38,39]. *Aspergillus flavus* is an opportunistic fungal plant and human pathogen because it produces mycotoxins, including aflatoxin B_1_, as well as other toxic secondary metabolites. In addition to infecting important crops, *A. flavus* also causes a deadly lung infection known as invasive aspergillosis. Although *A. flavus* is the second leading cause of this disease, after *Aspergillus fumigatus*, infections caused by *A. flavus* are 100-fold more virulent than those caused by *A. fumigatus* [40]. The two identified endophytic *Penicillium* species (*P. chermesinum*, *P. paxilli*) have multiple agricultural, biotechnological, and pharmaceutical applications, and might be interesting candidates for the isolation of antiparasitic agents or plant growth-promoting substances [41]. Ernst, Neubert [42] investigated niche partitioning of the two closely related fungal endophytes, *M. bolleyi* and *M. phragmitis*, which colonize *Phragmites australis*. Interestingly, the host habitat significantly differentiated the two species, whereas the latter one (also identified in this study) favors flooded regions. This fungus might be an indicator for the air moisture during fruit handling, storage, and transport. In summary, it is important for the food industry to know which fungi are associated with the passion fruits. Some of them might not cause disease symptoms in plants, but propagate in the harvested fruit and can produce toxins for humans. On the other hand, the beneficial microbes might restrict the spread of the pathogens, and the established equilibrium in the fungal populations might be important for the food quality.

### 4.1. Comparison of the Fruit of the Yellow and Purple Cultivar

The re-infection assays clearly demonstrated that the yellow fruit are more susceptible for pathogen infections than the purple fruit (Figure 4 and Figure 5). As expected, fruit with intact peels perform better than those in injured peels. Our results are similar with the previous report by Cerqueira-Silva, Jesus [43] in which the comparative results suggested that the purple passion fruit is resistant to woodiness virus. The comparative omics analyses of the peels of the two cultivars uncovered compounds, which might participate in, or are even responsible for, better resistance of the purple cultivar against pathogenic fungal infections.

### 4.2. Hormones

Analyses of the defense-related phytohormones concentrations suggest that they play different roles in the peels of the two cultivars. As expected, the SA and JA levels in the uninfected peels of both cultivars are quite low (Table 4). Since both hormones are induced upon pathogen infections, this result is not surprising, although several reports showed that fruit may have high SA levels to protect them against biotrophic pathogen attacks [44]. The oxylipin *cis*-OPDA is a precursor of jasmonates, but has also quite different defense signaling functions [45]. *cis*-OPDA can be esterified to galactolipids and the resulting compounds are thought to act as a rapidly available *cis*-OPDA source. Furthermore, *cis*-OPDA has been proposed to interact with ABA [46]. The elevated *cis*-OPDA level in the yellow peels may allow a faster activation of JA-dependent defense responses upon pathogen attack, the crosslink to ABA may promote lignin deposition in response to environmental stress [47]. Lignin deposition requires cell wall rearrangement, and the peels of ripening fruit undergo substantial softening, which require ABA-induced transcriptional changes in cell wall degrading enzymes [48]. As outlined by Forlani, Masiero [49], the multiple roles of ABA during fruit ripening makes it difficult to relate them to specific responses. In conclusion: comparison of both cultivars suggest that the yellow peels utilize more *cis*-OPDA and ABA for stress responses, and that elevated ABA level may also protect them better against abiotic stress [50].

### 4.3. Expression and Metabolite Profiles

An interesting observation is that about 1/3 of all genes are differentially expressed in the peels of the two cultivars and this pattern is more or less found for genes belonging to all biochemical pathways (Table 5 and Table S2). Consistent with the metabolite profiles, the KEGG analyses clearly shows that a huge number of genes for the phenylpropanoid and flavonoid pathways are much stronger expressed in the purple peels. The color of the fruit of the purple cultivar is caused by the anthocyanin cyanidin-3-O-glucoside. Together with peonidin-3-glucoside and the flavonoids, which are present in the purple fruits, these secondary metabolites clearly participate in plant defense against several stresses including pathogenic fungi [51]. Catechin, gallocatechin, and epicatechin have anti-oxidant and anti-inflammatory activities [52]. These results indicate that major metabolites, which are present in the purple, and reduced or missing in the yellow fruit, restricts pathogen growth on the fruits. The analysis of the DEGs also uncovered that those for antioxidant enzymes and proteins, receptors and signaling compounds for pathogen-associated molecular patterns, transporters predicted to be involved in detoxification and ion transport, redox regulators, and systemic signal propagation are much higher expressed in the purple peels (Figure 6 and Figure 7). Overall, the purple peels invest more in cell-protective functions and biotic stress responses.

## 5. Conclusions

This analyses uncovered pathogenic and non-pathogenic fungi, which are present on harvested passion fruits. Six pathogenic fungal species induce post-harvest rots; fruit of the yellow cultivar are more susceptible than the fruit of the purple cultivar. The comparative analyses of the peels suggest that flavonoids and phenylpropanoids might be responsible for the better resistance of the purple peels to decay development. The role of the non-pathogenic fungi requires further investigations. They might restrict pathogen growth, participate in strengthening the immunity of the harvested fruit, and might provide an important link between disease symptom development and pathogen resistance of the harvested fruits. The identified microorganisms as well as the obtained datasets for the fruit of the two cultivars provide a valuable source for future research on the control of postharvest passion fruit decay, which will be beneficial for the passion fruit industry.

## Figures and Tables

**Table 1:** Categorization of 26 isolates into 12 morphotypes based on morphological and ITS.

Morphotype	Number of Isolates	Passion Fruit Cultivar	Identified Species
1	3	yellow and purple	*Fusarium kyushuense*
2	3	yellow and purple	*Fusarium concentricum*
3	2	yellow	*Colletotrichum truncatum*
4	5	purple	*Alternaria alternata*
5	4	yellow and purple	*Cladosporium tenuissimum*
6	1	yellow	*Fusarium equiseti*
7	3	yellow and purple	*Aspergillus aculeatus*
8	1	yellow	*Aspergillus europaeus*
9	1	yellow	*Aspergillus flavus*
10	1	yellow	*Penicillium chermesinum*
11	1	purple	*Penicillium paxilli*
12	1	yellow	*Microdochium phragmitis*

**Table 2:** Morphological characteristics of 12 morphotypes of the isolated fungal species based on colony and conidial characters.

Morphotype	Colony on PDA Media		Conidia	
	Morphology	Growth Rate (mm/Week)	Length (µm)	Shape
Type-1	Reddish-white and floccose mycelia with deep red pigmentation on the agar side.	60–65	20–25	Obovate, ellipsoidal to clavate and 3–5 septate in macro-conidia.
Type-2	Reddish to white with lavish cottony mycelia. White to pale yellow pigmentation on the agar side.	50–55	26–45	Oval, obovoid to allantoid and macro-conidia slender with 3–5 septate.
Type-3	White grayish to dark grey, light to dark reverse pigmentation.	55–60	21–27	Non-septate, hyaline, falcate, and truncate.
Type-4	White to gray at the edge and olivaceous buff in the center.	55–60	25–56	Chains, obclavate, ovoid or ellipsoid and three to seven transverse septa.
Type-5	Olive-brown to dark green with gray-olivaceous to white edges, velvet-like texture with radially furrowed, dark pigmentation on the agar side.	35–40	7–12	Smooth, single-celled, olive-brown, elliptical to limonifor.
Type-6	Lavish white and fluffy aerial mycelium with dark to pale brown from front and pigments on the agar side.	27–35	13–34	Macro-conidia with mostly three to five-septae, slightly curved to lunate at apex.
Type-7	Dark brown to black colonies with rough texture, white mycelia underneath the colonies. Whitish yellow radial furrows at the backside.	30–35	4–5	Dark brown to black conidia, ellipsoidal, phialides, spinose, borne in radiate heads.
Type-8	Plane colonies, floccose from center with strong sporulation, no soluble pigment, and light olive on the agar side.	20–25	3–4.5	Globose conidia, roughened and yellow-brown to brown at maturity.
Type-9	Plain and flat at the edges, raised at the center and wrinkled cerebriform pattern, produce greenish conidia with a white border, cream color on the agar side.	20–25	3–6	Conidia with a thick mycelial mat, globose shape, thin walls and rough texture. Metulae obscured on the entire surface of the vesicles.
Type-10	Fast growing, white green-gray shade, dense conidiophores, and non-circular growth.	22–25	2.5–4	Basocatenate, hyaline or greenish, globose, ellipsoidal, cylindrical or fusiform, and smooth or rough-walled.
Type-11	Fast growing, white to light green shade, dense conidiophores with white edges and irregular growth.	18–25	2.5–4	Hyaline or greenish, globose, ellipsoidal, cylindrical, or fusiform.
Type-12	Pinkish white flat colonies, entire margins, slightly raised to umbonate center and greyish orange on the agar side.	35–40	-	No conidia produced in lab condition.

**Table 3:** Best BLAST matches for fungi based on ITS-rDNA regions.

Isolates	Morpho-Type	Accession Numbers ^a^	Fungal Taxon	Query Cover (%)	Seq. Similarity (%)	Accession Numbers of ITS-rDNA ^b^
YPF-1	Type-1	MW880893	*Fusarium kyushuense*	96	100	KC466546
PPF-1	Type-1	MW880906	*Fusarium kyushuense*	100	100	KC466546
PPF-2	Type-1	MW880907	*Fusarium kyushuense*	100	100	KC466546
YPF-2	Type-2	MW880894	*Fusarium concentricum*	100	99	MN341308
YPF-12	Type-2	MW880904	*Fusarium concentricum*	100	100	LC317601
PPF-8	Type-2	MW880913	*Fusarium concentricum*	100	100	LC317601
YPF-10	Type-3	MW880902	*Colletotrichum truncatum*	100	100	JQ936246
YPF-11	Type-3	MW880903	*Colletotrichum truncatum*	100	100	JQ936246
PPF-9	Type-4	MW880914	*Alternaria alternata*	100	100	MN547372
PPF-10	Type-4	MW880915	*Alternaria alternata*	100	100	MN547372
PPF-11	Type-4	MW880916	*Alternaria alternata*	100	100	MT482506
PPF-12	Type-4	MW880917	*Alternaria alternata*	100	100	MN547372
PPF-13	Type-4	MW880918	*Alternaria alternata*	100	100	MN547372
YPF-9	Type-5	MW880901	*Cladosporium tenuissimum*	100	100	MF422152
PPF-3	Type-5	MW880908	*Cladosporium tenuissimum*	100	100	MF422152
PPF-4	Type-5	MW880909	*Cladosporium tenuissimum*	100	100	MF422152
PPF-5	Type-5	MW880910	*Cladosporium tenuissimum*	100	100	MF422152
YPF-13	Type-6	MW880905	*Fusarium equiseti*	100	100	KR364600
YPF-3	Type-7	MW880895	*Aspergillus aculeatus*	99	99	KU203321
YPF-4	Type-7	MW880896	*Aspergillus aculeatus*	100	100	EU645733
PPF-7	Type-7	MW880912	*Aspergillus aculeatus*	100	100	LC514695
YPF-5	Type-8	MW880897	*Aspergillus europaeus*	100	100	FR727118
YPF-6	Type-9	MW880898	*Aspergillus flavus*	100	100	MG228413
YPF-7	Type-10	MW880899	*Penicillium chermesinum*	100	100	MK450679
PPF-6	Type-11	MW880911	*Penicillium paxilli*	100	98	AB933278
YPF-8	Type-12	MW880900	*Microdochium phragmitis*	99	97	AM502263

^a^ accession numbers of identified isolates in this study, ^b^ accession numbers of reference isolates from GenBank.

**Table 4:** Phytohormones levels in the peels of yellow and purple fruit cultivars.

Fruit Peels of Cultivar	SA (µg kg^−1^)	JA (µg kg^−1^)	JA-Ile (µg kg^−1^)	*cis*-OPDA (µg kg^−1^)	ABA (µg kg^−1^)
Purple	504 ± 81	34 ± 4	1.3± 0.3	3.1± 0.4	1133 ± 56
Yellow	449 ± 73	24 ± 4	2.2± 0.4	21.9 ± 3	2644 ± 94

The results are based on five independent experiments and units describes the amount of phytohormones per kg of fruit fresh weight. Errors are SEs. SA, salicylic acid; JA, jasmonic acid; JA-Ile, jasmonoyl-isoleucine; cis-OPDA, cis-12-oxophytodienoic acid; ABA, abscisic acid.

**Table 5:** Genes involved in various protection mechanisms with higher expression in the peels of the purple fruit in comparison to the yellow fruits. The data are based on three independent experiments.

Unigenes	Log_2_ Purple vs. Yellow	Gene Annotation
060661	3.2	ABC transporter C family member
073495	3.2	PLAT domain-containing protein
012452	3.2	leucoanthocyanidin reductase
066444	3.2	ABC transporter G family member
078965	3.2	Toll-like receptor
079007	3.2	ABC transporter B family member
157353	3.2	glutathione S-transferase
075520	3.3	disease resistance protein
066058	3.3	AP2-like ethylene-responsive transcription factor
014357	3.3	disease resistance protein
002704	3.5	RALF protein
075276	3.5	ethylene-responsive transcription factor
019034	3.6	LRR receptor-like serine/threonine-protein kinase
080936	3.6	LRR receptor-like serine/threonine-protein kinase
115445	3.6	cellulose synthase subunit
006373	3.6	allene oxide synthase
079086	3.6	ethylene-responsive transcription factor
013300	3.7	detoxification protein
072633	3.7	MLO-like protein
058972	3.7	ent-kaurene oxidase
065344	3.7	callose synthase
012846	3.7	disease resistance protein
077385	3.7	ethylene-responsive transcription factor
013346	3.8	salicylate carboxymethyltransferase
066699	3.9	mechanosensitive ion channel
081472	3.9	disease-resistance receptor-like protein kinase
045952	3.9	pathogenesis-related protein
081029	3.9	disease resistance protein
074186	3.9	detoxification protein
092433	3.9	ethylene-responsive transcription factor
076826	4.0	ethylene-responsive transcription factor
029255	4.0	remorin
099452	4.1	PLAT domain-containing protein
034958	4.1	flavonol synthase
005394	4.1	detoxification protein
058910	4.2	elicitor-responsive protein
151621	4.2	1-amino-cyclopropane-1-carboxylic acid oxidase
077400	4.2	respiratory burst oxidase homolog protein C
078602	4.3	Downy Mildew Resistance protein
080595	4.3	TMV resistance protein
153053	4.3	remorin
153975	4.3	1-aminocyclopropane-1-carboxylate oxidase
051447	4.5	ethylene-responsive transcription factor
073788	4.6	leucoanthocyanidin reductase
135950	4.7	pathogenesis-related genes transcriptional activator
077933	4.7	MLO-like protein
078748	4.7	ethylene-responsive element-binding protein
018995	4.7	monodehydroascorbate reductase
063867	4.8	ethylene-responsive transcription factor
004586	4.8	phenylalanine ammonia-lyase
081281	4.8	leucine-rich repeat receptor-like serine/threonine kinase
013377	4.8	4-coumarate-CoA ligase
080912	4.9	ABC transporter B family member
078836	4.9	EIN3 domain-containing protein
079312	4.9	ABC transporter G family member
078811	4.9	multidrug resistance P-glycoprotein
009128	5.0	ethylene receptor-like protein
058955	5.2	monoterpene synthase
079862	5.2	shikimate O-hydroxycinnamoyltransferase
076879	5.3	linoleate 13S-lipoxygenase
115915	5.5	disease resistance protein
017693	5.6	terpene synthase
080270	5.6	glutathione S-transferase
076381	5.6	ABC transporter G family member
147063	5.7	ethylene-responsive transcription factor
073453	5.9	flavonoid C-glucosyltransferase
141905	6.5	chalcone-flavonone isomerase
077293	6.7	terpene synthase
065968	7.1	LRR receptor-like serine/threonine-protein kinase
074648	7.1	ethylene-responsive transcription factor
058571	7.6	flavonoid hydroxylase
073617	7.6	remorin
134682	7.7	leucoanthocyanidin reductase
063666	8.0	sieve element occlusion protein
065392	8.2	caffeoyl-CoA O-methyltransferase
081612	8.3	malonyl-CoA:anthocyanidin 5-O-glucoside
006561	8.8	naringenin-chalcone synthase
148145	9.4	glutathione S-transferase
079417	9.7	phenylalanine ammonia-lyase
015737	12.0	glutathione S-transferase
051561	12.5	naringenin,2-oxoglutarate 3-dioxygenase
079297	12.8	leucoanthocyanidin dioxygenase

**Table 6:** Mean peak intensities for metabolites partially identified in peels of the purple and yellow passion fruit cultivars by LC-ESI-Q-ToF-MS. (cf. Methods and Materials). Metabolites with highest intensities and differences between the purple and yellow cultivars are shown. Based on three independent experiments. Compounds are listed in order of retention time.

Peak No.	Molecular Formula	m/z Measured and Ionization Mode	Mean Intensity Purple	Mean Intensity Yellow	t-Test	Identification
1	C_5_H_10_N_2_O_3_	147.0765-pos	115,676	192,815	0.000	glutamine
2	C_6_H_12_O_6_	219.02657-pos	102,721	56,375	0.000	glucose
3	C_5_H_9_N_1_O_4_	148.06045-pos	30,462	33,943	0.002	glutamic acid
4	C_17_H_31_N_1_O_15_	490.17676-pos	26,190	32,155	0.001	unknown
5	C_12_H_22_O_11_	341.1089-neg	100,948	93,073	0.092	sucrose
6	C_6_H_8_O_6_	175.02481-neg	119,067	65,947	0.001	ascorbic acid
7	C_4_H_6_O_5_	133.01433-neg	164,764	153,107	0.028	malic acid
8	C_8_H_8_O_7_	191.01975-neg	154,828	18,986	0.000	citric acid
9	C_10_H_17_N_3_O_6_S	308.0911-pos	59,473	48,847	0.035	glutathione
10	C_10_H_13_N_5_O_4_	268.10409-pos	220,255	301,724	0.002	adenosine
11	C_5_H_4_O_4_	129.01819-pos	21,815	7486	0.000	unknown
12	C_5_H_9_N_1_O_2_	116.0705-pos	3322	3144	0.445	proline
13	C_9_H_11_N_1_O_3_	182.08121-pos	10,101	8574	0.323	tyrosine
15	C_5_H_7_N_1_O_3_	130.04991-pos	219,080	63,362	0.000	5-oxoproline
16	C_8_H_10_N_1_	166.0862-pos	16,439	69,894	0.000	phenylalanine
17	C_15_H_18_N_4_O_11_	431.1048-pos	56,678	6094	0.001	unknown
18	C_15_H_14_O_7_	305.06663-neg	39,016	0	0.000	gallocatechin
19	C_22_H_22_O_11_	463.12378-pos	296,537	578	0.000	peonidin-3-glucoside
20	C_15_H_14_O_6_	291.08636-pos	55,091	3370	0.000	catechin
21	C_21_H_20_O_11_	449.10812-pos	114,061	2179	0.000	cyanidin-3-O-glucoside
22	C_13_H_18_O_8_	301.09273-neg	54,632	2111	0.000	unknown
23	C_26_H_34_O_12_	537.1979-neg	9365	5135	0.001	citrusin A/hyuganoside III
24	C_21_H_22_O_11_	449.10839-neg	96,753	0	0.000	unknown flavonoid
25	C_21_H_22_O_11_	451.12317-pos	94,078	0	0.000	unknown flavonoid
26	C_26_H_34_O_12_	537.19771-neg	37,255	30,798	0.048	citrusin A/hyuganoside III
27	C_15_H_14_O_6_	291.08639-pos	105,416	0	0.000	epicatechin
28	C_20_H_27_N_1_O_10_	440.1564-neg	278,197	173,262	0.005	prunasin-rhamnoside
29	C_14_H_17_N_1_O_6_	296.1128-pos	45,051	45,072	0.993	prunasin
30	C_21_H_20_O_11_	447.09301-neg	7203	131,254	0.000	C-glycosidic flavonoid
31	C_19_H_28_O_10_	415.1602-neg	1437	0	0.000	unknown
32	C_27_H_30_O_14_	579.17129-pos	299,342	0	0.000	unknown flavonoid
33	C_27_H_30_O_16_	609.14553-neg	125,726	1676	0.000	unknown flavonoid
34	C_15_H_12_O_7_	303.05087-neg	32,535	0	0.000	unknown
35	C_26_H_32_O_11_	521.2031-neg	22,200	7319	0.000	unknown
36	C_21_H_20_O_12_	463.08775-neg	97,156	0	0.000	unknown flavonoid
37	C_16_H_20_O_9_	395.07389-pos	26,622	49,305	0.117	unknown
38	C_21_H_20_O_10_	431.09818-neg	622	30,081	0.000	unknown
39	C_17_H_19_N_1_O_9_	382.11328-pos	274,985	470,037	0.000	prunasin malonate
40	C_27_H_30_O_13_	561.16079-neg	5768	129,117	0.000	unknown
41	C_27_H_28_O_14_	575.14075-neg	82,622	1955	0.001	unknown flavonoid
42	C_21_H_20_O_10_	433.11323-pos	115,448	941	0.000	unknown

## Data Availability

The pathogen sequence datasets were analyzed in this study and deposited to GenBank under accession number MW880893-MW880918.

## Supplementary Materials

The following are available online at https://www.mdpi.com/article/10.3390/jof7100879/s1, Figure S1: Morphological characterization of the 26 isolates collected from passionfruit postharvest, Table S1: GenBank accession numbers of sequences used for phylogenetic analyses. Table S2: Number of DEGs of all genes for the annotated pathways in the peels of the fruits of the purple and yellow passion fruit cultivars.
