# Supplementary material for: Identification of Fungal Pathogens to Control Postharvest Passion Fruit (Passiflora edulis) Decays and Multi-Omics Comparative Pathway Analysis Reveals Purple Is More Resistant to Pathogens than a Yellow Cultivar
Source: J Fungi (Basel). 2021 Oct 19;7(10):879. doi: 10.3390/jof7100879 (PMC8538400; doi:10.3390/jof7100879)
Supplement: Supplementary file 1 [file jof-07-00879-s001.zip › jof-1413052-supplementary.pdf]

Supplementary Material

Identification of fungal pathogens to control postharvest passion fruit (*Passiflora edulis*) decays and multi-omics comparative pathway analysis reveals purple is more resistant to pathogens than a yellow cultivar

Hafiz Muhammad Rizwan[1], Lin Zhimin[2], Wiwiek Harsonowati[3], Abdul Waheed[4], Yang Qiang[1], Ahmed Fathy Yousef[1,5], Nigarish Munir[1], Xiaoxia Wei[6], Sandra S Scholz[7], Michael Reichelt[8], Ralf Oelmüller[1,7], Faxing Chen[1,*]

*Correspondence: fxchen@fafu.edu.cn

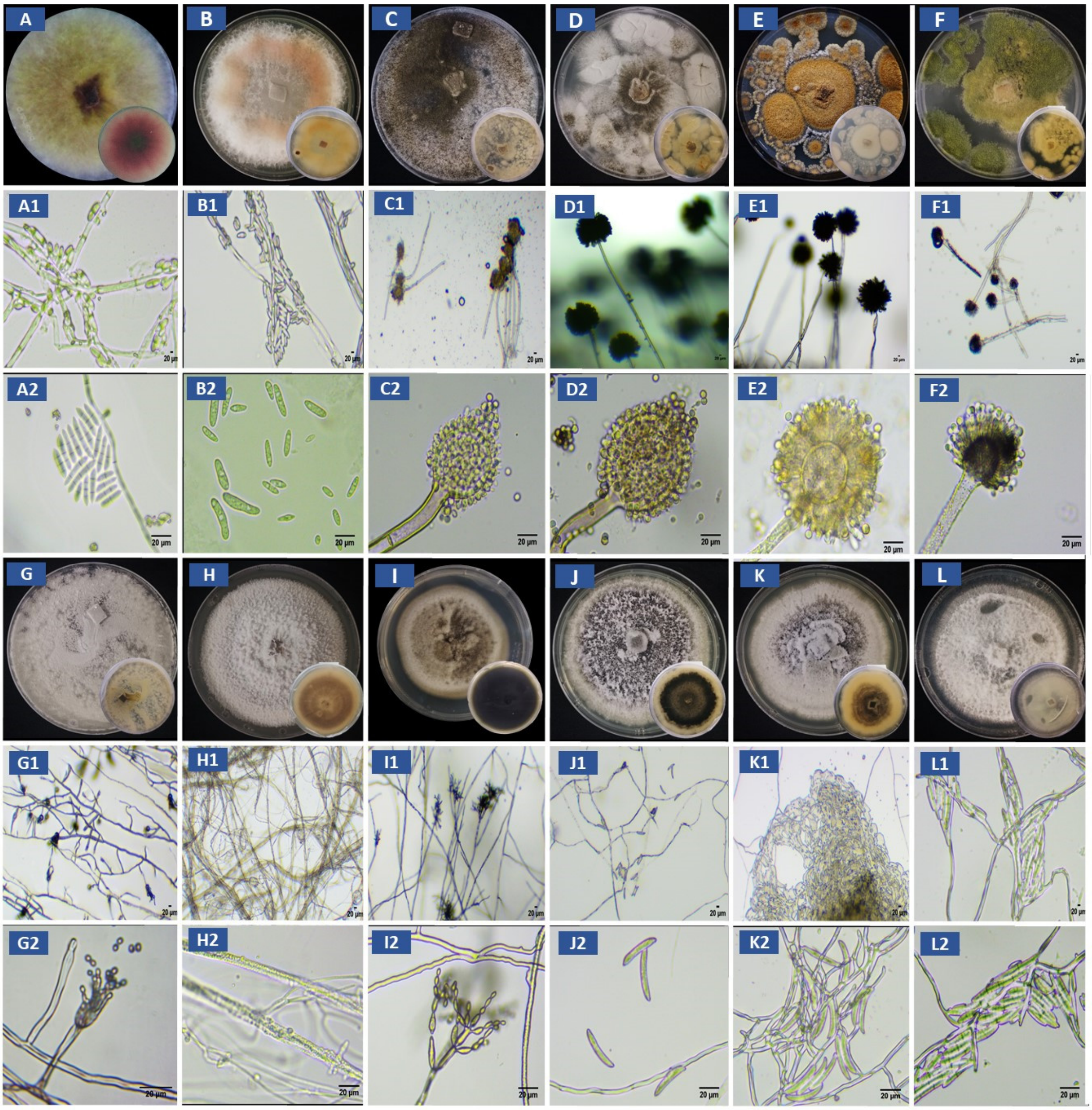

Figure S1. Continue to next page….

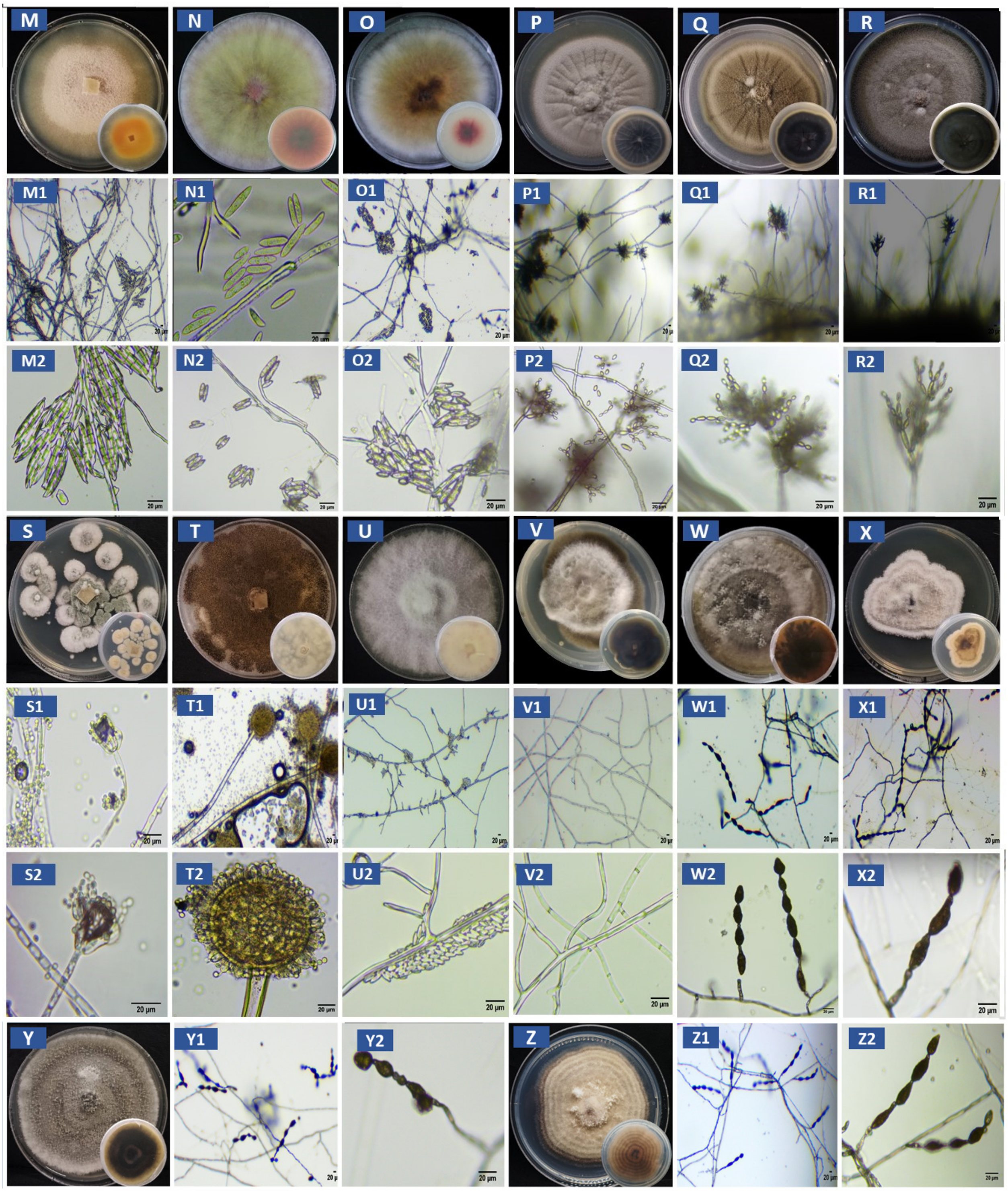

M
N
O
P
Q
R
M1
N1
O1
P1
Q1
R1
M2
N2
O2
P2
Q2
R2
S
T
U
V
W
X
S1
T1
U1
V1
W1
X1
S2
T2
U2
V2
W2
X2
Y
Y1
Y2
Z
Z1
Z2

Figure. S1: Morphological characterization of the 26 isolates collected from passionfruit postharvest rot grew on potato dextrose agar (PDA) for 10 days. Characteristics of colony morphology on PDA (A-Z), thread like structure showing hyphae and mycelium (a1-z1) conidia (a2-z2). Following are the isolates details A, a1 & a2= YPF-1 (*Fusarium kyushuense*); B, b1 &b2 = YPF-2 (*Fusarium concentricum*); C, c1 & c2 = YPF-3 (*Aspergillus aculeatus*); D, d1 & d2 = YPF-4 (3 (*Aspergillus aculeatus*); E, e1& e2= YPF-5 (*Aspergillus europaeus*); F, f1 & f2= YPF-6 (*Aspergillus flavus*); G, g1 & g2= YPF-7 (*Penicillium chermesinum*); H, h1 & h2 = YPF-8 (*Microdochium phragmitis*); I, i1 & i2 = YPF-9 (*Cladosporium tenuissimum*); J, j1 & j2= YPF-10 (*Colletotrichum truncatum*); K, k1 & k2 = YPF-11 (*Colletotrichum truncatum*); L, l1 & l2= YPF-12 (*Fusarium concentricum*); M, m1 & m2 = YPF-13 (*Fusarium equiseti*); N, n1 & n2= PPF-1 (*Fusarium kyushuense*); O, o1 & o2= PPF-2 (*Fusarium kyushuense*); P, p1 & p2= PPF-3 (*Cladosporium tenuissimum*); Q, q1 & q2= PPF-4 (*Cladosporium tenuissimum*); R, r1& r2 = PPF-5 (*Cladosporium tenuissimum*); S, s1 & s2= PPF-6 (*Penicillium paxilli*); T, t1 & t2= PPF-7 (*Aspergillus aculeatus*); U, u1 & u2= PPF-8 (*Fusarium concentricum*); V, v1& v2= PPF-9 (*Alternaria alternata*), W, w1 & w2= PPF-10 (*Alternaria alternata*); X, x1 & x2= PPF-11 (*Alternaria alternata*); Y, y1& y2= PPF-12 (*Alternaria alternata*); Z, z1 & z2= PPF-13 (*Alternaria alternata*); (bar = 20μm).

Table S1. GenBank accession numbers of sequences used for phylogenetic analyses.

Sr.	Genus	Species	Voucher/Strain	SSU+ITS+LSU
1	*Fusarium*	*kyushuense*	S8	MH892849
2	*Fusarium*	*kyushuense*	WZ-201	MN856334
3	*Fusarium*	*equiseti*	6mpc	HM060593
4	*Fusarium*	*equiseti*	JG22	KJ412501
5	*Fusarium*	*incarnatum*	PaB-3	MN6462580
6	*Fusarium*	*oxysporum*	MAFF 744009	DQ452448
7	*Fusarium*	*redolensene*	-	HQ443207
8	*Fusarium*	*concentricum*	GF-6	MT940455
9	*Fusarium*	*concentricum*	F2	HQ379633
10	*Fusarium*	*concentricum*	F21	HQ379647
11	*Colletotrichum*	*queenslandicum*	BRIP 57981a	KF877315
12	*Colletotrichum*	*brasiliense*	CBS:128528	JQ005234
13	*Colletotrichum*	*truncatum*	C-82	KY399773
14	*Colletotrichum*	*truncatum*	OCC69	KP743405
15	*Colletotrichum*	*truncatum*	OCC98	KP743406
16	*Microdochium*	*phragmitis*	GBC-Fungus 115	MN077485
17	*Microdochium*	*phragmitis*	SL2-5A	MN328308
18	*Cladosporium*	*tenuissimum*	COAD	MH682173
19	*Cladosporium*	*tenuissimum*	CBS 262.80	HM148201
20	*Cladosporium*	*tenuissimum*	HLGM02	MN005091
21	*Aspergillus*	*aculeatus*	UASWS0464	HQ166575
22	*Penicillium*	*chermesinum*	NOU279	MN220649
23	*Penicillium*	*chermesinum*	CMV011D8	MK450679
24	*Penicillium*	*paxilli*	PP12	FJ884120
25	*Penicillium*	*paxilli*	A71	DQ480346
26	*Aspergillus*	*europaeus*	SM03	MW145184
27	*Aspergillus*	*europaeus*	-	FR727118
28	*Aspergillus*	*flavus*	GFRS16	MT447484
29	*Aspergillus*	*flavus*	EF 544	MT529193
30	*Alternaria*	*alternata*	m110801-3-3	KC816019
31	*Alternaria*	*alternata*	CATAS-AA01	GQ169766
32	*Alternaria*	*alternata*	CBS 630.93	KJ718210
33	*Alternaria*	*alternata*	PaF-3	MN646267
34	*Ganoderma*	*boninense*	GB001	KX092000

Table S2. Number of DEGs of all genes for the annotated pathways in the peels of the fruits of the purple and yellow passion fruit cultivars.

Pathway	ID	DEG	all genes
Carbon metabolism	ko01200	169	458
Biosynthesis of amino acids	ko01230	164	417
**Plant hormone signal transduction**	**ko04075**	**127**	**323**
Starch and sucrose metabolism	ko00500	111	285
Protein processing in endoplasmic reticulum	ko04141	107	275
Spliceosome	ko03040	106	247
Endocytosis	ko04144	104	249
Ribosome	ko03010	88	376
Purine metabolism	ko00230	82	263
RNA transport	ko03013	81	261
Glycolysis / Gluconeogenesis	ko00010	77	202
Plant-pathogen interaction	ko04626	77	197
Phenylpropanoid biosynthesis	ko00940	72	142
Cysteine and methionine metabolism	ko00270	70	164
Ubiquitin mediated proteolysis	ko04120	68	175
Peroxisome	ko04146	68	164
Amino sugar and nucleotide sugar metabolism	ko00520	63	194
RNA degradation	ko03018	63	166
Pyrimidine metabolism	ko00240	61	195
mRNA surveillance pathway	ko03015	57	187
Pyruvate metabolism	ko00620	57	149
Carbon fixation in photosynthetic organisms	ko00710	53	107
Fatty acid metabolism	ko01212	53	157
Phagosome	ko04145	52	132
Glyoxylate and dicarboxylate metabolism	ko00630	52	127
Oxidative phosphorylation	ko00190	50	205
Glycine, serine and threonine metabolism	ko00260	49	126
Glycerophospholipid metabolism	ko00564	48	141
Glutathione metabolism	ko00480	45	116
alpha-Linolenic acid metabolism	ko00592	45	97
2-Oxocarboxylic acid metabolism	ko01210	43	110
Glycerolipid metabolism	ko00561	42	103
Fatty acid degradation	ko00071	42	103
Phenylalanine metabolism	ko00360	41	70
Citrate cycle	ko00020	40	104
Valine, leucine and isoleucine degradation	ko00280	39	98
Phosphatidylinositol signaling system	ko04070	38	89
Ribosome biogenesis	ko03008	37	110
Phenylalanine, tyrosine and tryptophan biosynthesis	ko00400	37	77
Inositol phosphate metabolism	ko00562	37	94
beta-Alanine metabolism	ko00410	36	84
Cyanoamino acid metabolism	ko00460	35	68
Arginine and proline metabolism	ko00330	35	92
Terpenoid backbone biosynthesis	ko00900	34	84
Alanine, aspartate and glutamate metabolism	ko00250	33	89
Aminoacyl-tRNA biosynthesis	ko00970	33	112
Sulfur metabolism	ko00920	32	69
Nucleotide excision repair	ko03420	32	95
Pentose and glucuronate interconversions	ko00040	32	77
Galactose metabolism	ko00052	32	79
Tyrosine metabolism	ko00350	32	63

Pentose phosphate pathway	ko00030	32	89
Ubiquinone and other terpenoid-quinone biosynthesis	ko00130	30	61
Propanoate metabolism	ko00640	29	73
Porphyrin and chlorophyll metabolism	ko00860	28	74
Arginine biosynthesis	ko00220	28	75
N-Glycan biosynthesis	ko00510	27	73
Proteasome	ko03050	27	85
Basal transcription factors	ko03022	27	66
Fatty acid biosynthesis	ko00061	26	73
Ascorbate and aldarate metabolism	ko00053	25	59
Photosynthesis	ko00195	25	61
Fructose and mannose metabolism	ko00051	25	80
Circadian rhythm - plant	ko04712	25	59
Protein export	ko03060	25	79
Regulation of autophagy	ko04140	24	57
Nitrogen metabolism	ko00910	23	56
Biosynthesis of unsaturated fatty acids	ko01040	23	80
Pantothenate and CoA biosynthesis	ko00770	22	56
**Flavonoid biosynthesis**	**ko00941**	**22**	**29**
Tropane, piperidine and pyridine alkaloid biosynthesis	ko00960	20	44
DNA replication	ko03030	20	69
Sphingolipid metabolism	ko00600	20	51
Homologous recombination	ko03440	20	69
Steroid biosynthesis	ko00100	19	41
Other glycan degradation	ko00511	19	46
Tryptophan metabolism	ko00380	19	50
Base excision repair	ko03410	19	60
Carotenoid biosynthesis	ko00906	19	39
Lysine degradation	ko00310	18	52
SNARE interactions in vesicular transport	ko04130	18	53
RNA polymerase	ko03020	18	67
Isoquinoline alkaloid biosynthesis	ko00950	16	31
Mismatch repair	ko03430	16	55
Valine, leucine and isoleucine biosynthesis	ko00290	16	36
ABC transporters	ko02010	16	43
Linoleic acid metabolism	ko00591	15	29
Fatty acid elongation	ko00062	15	37
Histidine metabolism	ko00340	14	37
Selenocompound metabolism	ko00450	14	33
Nicotinate and nicotinamide metabolism	ko00760	14	40
Ether lipid metabolism	ko00565	12	41
Taurine and hypotaurine metabolism	ko00430	11	21
Stilbenoid, diarylheptanoid and gingerol biosynthesis	ko00945	11	17
Glycosylphosphatidylinositol(GPI)-anchor biosynthesis	ko00563	11	33
Diterpenoid biosynthesis	ko00904	11	25
Brassinosteroid biosynthesis	ko00905	11	20
Monoterpenoid biosynthesis	ko00902	8	13
Sesquiterpenoid and triterpenoid biosynthesis	ko00909	6	11
Cutin, suberine and wax biosynthesis	ko00073	6	20
Glycosphingolipid biosynthesis	ko00604	5	12
Lipoic acid metabolism	ko00785	3	9
Flavone and flavonol biosynthesis	ko00944	2	2
Glucosinolate biosynthesis	ko00966	1	2
Limonene and pinene degradation	ko00903	1	5
